# Effect of Cyclosporine-A on Paraoxonase Activity in Wistar Rats

**Published:** 2011-02-01

**Authors:** H. Argani, A. Ghorbanihaghjo, N. Rashtchizadeh, S. Seifirad, Y. Rahbarfar

**Affiliations:** 1 *Drug Applied Research Center, Tabriz University of Medical Sciences, Tabriz, Iran.*; 2 *Biotechnology reaserch center, Tabriz University of Medical Sciences, Tabriz, Iran*; 3 *Nephrology Division, Modarres Hospital, Shahid Beheshti University of Medical Sciences, Tehran, Iran. *

**Keywords:** Cyclosporine A, Paraoxonase, Total antioxidant, malondialdehyde

## Abstract

Background: Many adverse effects have been reported on using cyclosporine (CSA) in organ transplantation.

Objective: To investigate the effects of CSA on paraoxonase (PON) activity and lipid peroxidation metabolites in early and late-stage of peroxidation and also total antioxidant (TA).

Methods: Twenty 220-250 g adult male Wistar rats were included in the study. The animals were stored for one week in the animal room before the initial injection to habituate with temperature, humidity, and circadian rhythm of day (12 h) and night (12 h). The temperature was kept at 23 °C. Animals had access to food and water *ad libitum*.

Results: A significant (p=0.002) increase in the serum levels of conjugated diones was observed in the case compared to the control group. At the end of the study, malondialdehyde (MDA) levels in CSA group was significantly (p=0.01) higher than the control group. Serum PON1 activity was significantly (p=0.004) lower in the case than the control group.

Conclusion: CSA administration could impair oxidant-antioxidant pathways and increase oxidative stress. Antioxidant therapy could be beneficial in patients treated with CSA.

## INTRODUCTION

Cyclosporine-A (CSA) is one of the most effective immunosuppressive drugs which has been used in autoimmune diseases and prevention of liver and kidney transplant rejections [1-3]. Although CSA is known as the drug of choice in transplantation [4], many adverse effects have been reported with this cytotoxic agent. Side effects include nephrotoxicity, damage to the structure of lipoproteins, atrophy of renal tubules, interstitial renal tissue fibrosis, progressive renal damage [1-3], hypertension [5], contraction of afferent renal vessels and decreased glomerular filtration rate [4]. It has been demonstrated that CSA inhibits nitric oxide (NO) synthesis and activates protein kinase C (PKC) and production of thromboxane A2 (TBXA2) [6]. Although many studies tried to explain the mechanism of CSA adverse effects, the underlying mechanism for CSA side effects has still remained unclear [2]. Most of the published studies, however emphasize on molecular and cellular damage caused by increased production of free radicals as the underlying mechanism of CSA toxicity [7,8]. In fact, oxidative stress resulting from administration of CSA is known as the main cause of adverse effects such as atherosclerosis, thus antioxidant therapy in patients receiving CSA are considered in many studies. Spironolactone, calcium channel blockers and antioxidant drugs such as ramipril, trimetazicine, allopurinol and melatonin have been tried to decrease this adverse effects and reported favorable results [9-11].

It has been demonstrated that CSA reduces activity of antioxidant enzymes such as superoxide dismutase (SOD), catalase and glutathione peroxidase. Based on this knowledge, scavenger antioxidants such as vitamin E, melatonin and N-acetyl-cysteine have been studied to reduce CSA nephropathy. But role of paraoxonase (PON) as an antioxidant enzyme has not been adequately studied [12-14].

PON1 are a group of enzymes which hydrolyze organophosphates in cellular pathways [15]. They have been divided into three genetic isoforms. Their genes have been located on long arm of 7^th^ chromosome, q7 [16]. PON1 is a protein consisted of 354 amino acids and has a molecular weight of 43 kDa, which is produced by hepatic cells and banded and transferred with high-density lipoprotein (HDL-C) in plasma. It is located on Apo-1 part of HDL-C as an antioxidant which prevents LDL-C oxidation [17]. The serum level of PON1 is affected by inflammatory changes and oxidized LDL-C (OX-LDL) levels, thus this molecule is responsible for HDL-C anti-atherosclerotic effects [15]. Activity of PON1 is influenced by genetic and environmental factors. Environmental factors which affecting PON1 activity include cigarette smoking, obesity and vitamin absorption [18].

PON is one of the most powerful enzymes for removing free radicals, and protecting lipoproteins against oxidant agents. PON hydrolyze organophosphates such as paraoxone and aromatic esters such as phenyl acetate [19]. A negative correlation between PON activity and oxidative stress in serum and macrophages is shown [20]. Reduced PON activity has been detected in patients with increased oxidative stress such as patients with hyperglycemia, diabetes, and hypercholesterolemia, and patients who suffer from cardiovascular disease [21,22]. PON inhibits LDL oxidation and plays a key role in protecting LDL from oxidation and preventing lipoperoxides accumulation. It has been shown that decreased activity of PON is associated with increased LDL oxidation, foam cells accumulation in arterial walls, and increased atherosclerosis [23]. It has been shown that PON activity decreased in dialysis patients. Several studies investigated the effects of drugs on PON activity and reported positive effects of simvastatin on PON activity and nandrolone decanonate effects in reduction of PON activity [24,25]. To the best of our knowledge, few studies have investigated the effects of CSA on PON activity. This study was therefore undertaken to investigate the effects of CSA on PON activity and lipid peroxidation metabolites in early stages of peroxidation, including conjugated dienes (CD), and late-stage of peroxidation including malondialdehyde (MDA) and total antioxidant (TA). The possible correlation between this parameters was also considered.

## MATERIAL AND METHODS

Animals

Twenty 220–250 g adult male Wistar rats were included in the study. The animals were stored for one week in the animal room before the initial injection to habituate with temperature, humidity, and circadian rhythm of day (12 h) and night (12 h). The temperature was kept at 23 °C. Normal food and water was available in the animals’ cage within the whole time of the study.

Methodology

Twenty Wistar rats were randomized into two groups. Group A rats (case), received subcutaneous CSA (15 mg/kg) daily for 14 days. Group B rats (control) received normal saline injections. On the 15th day of the study, blood samples were collected through orbital sinus using capillary tubes. Each sample was then divided into two equal aliqoutes of 0.5 mL of whole blood—one in EDTA to measure CSA level, and another to be centrifuged; the serum was kept in Epindorf vials and stored less than two days at 70 °C freezer before other laboratory analysis.

Laboratory analysis

Serum PON1 and aryl activity was determined spectrophotometrically using paraoxon (O, O-diethyl-O-P-nitrophenylphosphate) and phenylacetate as substrates, respectively [26]. MDA level, as an index of lipid peroxidation, was measured by the thiobarbituric acid (TBA) method [27]. Serum total antioxidant capacity (TA) was determined spectrophotometrically using Randox diagnostic kit.

Statistical analysis

SPSS® 16 for Windows® program was used to perform statistical analyses. Results are expressed as mean±SD. Mann-Whitney U test was used to assess the differences between the results of nonparametric continuous variables. Pearson’s correlation coefficient was used to evaluate the level of associations between two continuous variables. A p value <0.05 was considered statistically significant.

## RESULTS

Serum levels of HDL, TA, CD, MDA, and serum PON1 activity of case and control groups at the end of the study are shown in Table 1.

**Table 1:** Laboratory findings of the rats after treatment with CSA *vs*. placebo

**Variable**	**Placebo group (n=8)Mean**±**SD**	**CSA group (n=10) Mean**±**SD**	**p value** a
HDL (mg/dL)	26.7 1.11	25.8±1.40	*0.158*
LDL (mg/dL)	51.6 ± 4.75	87.26 ± 152.32	0.478
TA (mg/dL)	1.74 ± 0.16	1.51±0.19	*0.16*
PON (U/mL)	106.51 ± 10.06	92.67±7.36	*0.004*
MDA (nmol/mL)	1.79±0.15	2.16±0.19	0.01
CD (mg/dL)	2.44±0.13	2.83±0.19	*0.002*
Cholesterol (mg/dL)	88.5±5.40	86.6±4.17	*0.411*
TG (mg/dL)	51±4.04	53.60±3.77	*0.178*

HDL: High-density lipoprotein; LDL: Low-density lipoprotein; TA: Toyal antioxidant; PON: Paraoxonase; MDA: Malondialdehyde; CD: Congugated diens; TG: Triglycerides

a CSA treated group *vs*. placebo group

The mean±SD serum CSA level in case and control groups was 3371.13±44.9 and 0.00±0.0 ng/mL, respectively.

At the end of study, a significant (p=0.002) increase in the serum levels of CD was observed in the case compared to the control group.

Serum level of MDA, as a marker for peroxidation, was measured to assess the effect of CSA therapy on lipid peroxidation. At the end of study, MDA levels in CSA group was significantly (p=0.01) higher than the control group.

No statistically significant difference in the concentration of HDL was observed between the case and control groups at the end of the study. The mean±SD serum LDL level of case (152.32±87.26 mg/dL) and control group (51.6±4.75 mg/dL) was not statistically different at the end of the study. There were no significant differences in the serum concentrations of TG and cholesterol after CSA administration in the case and control groups. No statistically significant (p=0.16) difference was observed in the serum TA levels between the case and control group.

The effect of CSA administration on serum PON1 activity, an antioxidant enzyme, was also measured besides the above-mentioned parameters. At the end of the study, serum PON1 activity was significantly (p=0.004) lower in the case than the control group.

No statistical correlation was observed between PON activity and serum level of CSA (r=0.293, p=0.370).

**Figure 1:**
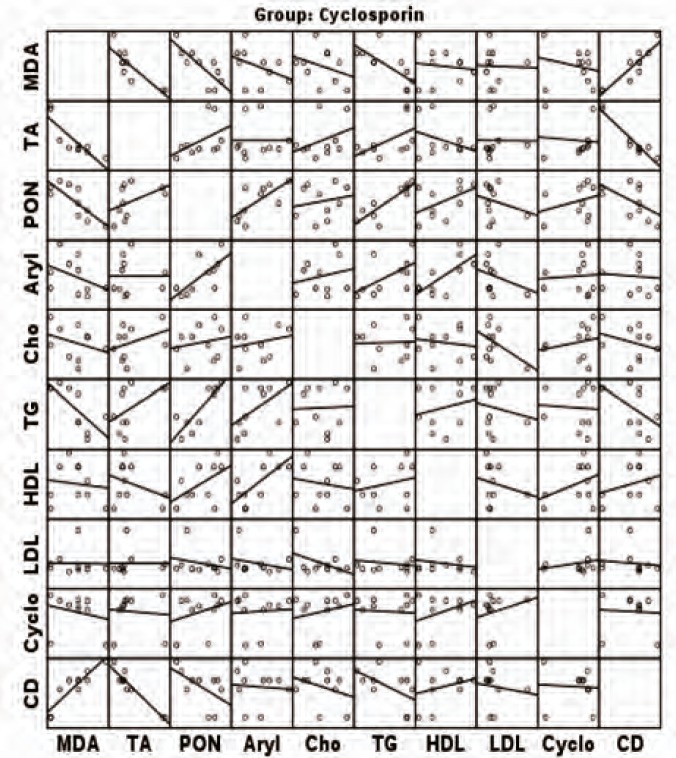
Correlation between measured parameters at the end of the study in the CSA group

**Figure 2:**
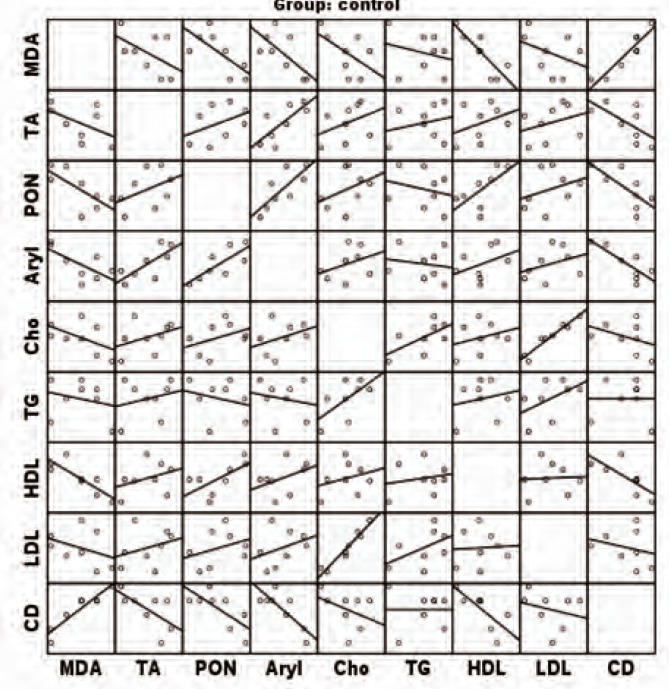
Correlation between measured parameters at the end of the study in the control group

Figures 1 and 2 show correlations between the measured parameters at the end of the study in CSA and control groups, respectively.

## DISCUSSION

This study was performed to assess the effects of CSA on PON activity and lipid peroxidation metabolites in early stages of peroxidation, including CD, and late-stage of peroxidation including MDA and TA. The possible correlation between these parameters was also assessed.

According to the results of our study, CSA administration tends to significantly decrease PON activity and TAS. A significant increase in MDA and CD serum levels, as lipid peroxidation markers, was also observed after CSA administration. The observed change in lipid profile (TG, total cholesterol, LDL, and HDL) was not statistically significant.

Although the oxidative stress was noticed in published studies as the main probable mechanism of CSA toxicity, the details of this process and the effect of CSA administration on known antioxidant particles was not well studied. It has been shown that oxygen free radicals play a key role in CSA cytotoxity [1-7].

Paragh, *et al*, in 1999 and Dantoine, *et al*, in 1998, compared PON activity in uremic patients, kidney transplant recipients (treated by CSA) and healthy subjects. Their results were similar to our observations. PON activity was significantly lower in their uremic patients but there was no significant difference in PON activity of kidney transplant recipients in comparison to healthy population. According to the results of these studies, CSA administration has no effect on PON activity. None of the above studies however, evaluated the effect of CSA on PON activity directly. Their population was kidney transplant recipients with impaired and complex oxidant-antioxidant pathways [26].

No statistically significant correlation was observed between serum level of CSA and PON activity in our study. Considering these results, we could conclude that CSA may decrease PON activity via a dose-independent mechanism and that it may tend increase the oxidative stress. Further studies with larger population size and also human studies are needed to approve this hypothesis.

Origlia, *et al*, have shown that L-Carnitin, as an antioxidant agent, could decrease CSA nephrotoxicity and lipid peroxidation. Results of their study emphasize that oxidative stress plays a key role in CSA cytotoxity [28]. A decrease in liver antioxidant capacity after CSA administration was also noticed by Durak, *et al*, and Ghaznavi, *et al* [29,30]. It has also been shown by Kim, *et al*, that TA levels after CSA administration in Wistar rats were decreased significantly [31]. Additionally, MDA levels was increased after CSA administration in Wistar rats. Results of our study on TAS and MDA are in keeping with the studies mentioned above.

The CD level in our study was also increased after CSA treatment. CD is a marker for early-stage lipid peroxidation; therefore with respect to the increased level of CD accompanied by increased level of MDA, a marker of late-stage lipid peroxidation, the results of this study emphasize the role of lipid peroxidation on CSA cytotoxicity.

It has been shown that hyperlipidemia is a common side-effect of CSA therapy in human subjects [32]. Wierzbika, *et al*, in their study on 35 liver transplanted children evaluated the effect of CSA therapy on serum lipid profile. They showed that only serum LDL and total cholesterol levels are increased significantly after CSA therapy. TG, HDL, and VLDL levels were not changed significantly [33].

Results of our study on changes in the lipid profile after CSA treatment are not in accord to some published studies. Short period of our study could probabl explain these results. It has been shown that change in lipid profile usually occurs after 3-5 years of CSA treatment [34,35].

In conclusion, CSA administration could impair oxidant-antioxidant pathways and increase oxidative stress. With respect to the results of our study, antioxidant therapy could be beneficial in patients treated with CSA.
